# Multisensor Analysis of Spectral Dimensionality and Soil Diversity in the Great Central Valley of California

**DOI:** 10.3390/s18020583

**Published:** 2018-02-14

**Authors:** Daniel Sousa, Christopher Small

**Affiliations:** Lamont-Doherty Earth Observatory, Columbia University, Palisades, NY 10964, USA; csmall@columbia.edu

**Keywords:** soil, spectral dimensionality, spectral resolution, spatial scaling, Great Central Valley, California, AVIRIS, Landsat

## Abstract

Planned hyperspectral satellite missions and the decreased revisit time of multispectral imaging offer the potential for data fusion to leverage both the spectral resolution of hyperspectral sensors and the temporal resolution of multispectral constellations. Hyperspectral imagery can also be used to better understand fundamental properties of multispectral data. In this analysis, we use five flight lines from the Airborne Visible/Infrared Imaging Spectrometer (AVIRIS) archive with coincident Landsat 8 acquisitions over a spectrally diverse region of California to address the following questions: (1) How much of the spectral dimensionality of hyperspectral data is captured in multispectral data?; (2) Is the characteristic pyramidal structure of the multispectral feature space also present in the low order dimensions of the hyperspectral feature space at comparable spatial scales?; (3) How much variability in rock and soil substrate endmembers (EMs) present in hyperspectral data is captured by multispectral sensors? We find nearly identical partitions of variance, low-order feature space topologies, and EM spectra for hyperspectral and multispectral image composites. The resulting feature spaces and EMs are also very similar to those from previous global multispectral analyses, implying that the fundamental structure of the global feature space is present in our relatively small spatial subset of California. Finally, we find that the multispectral dataset well represents the substrate EM variability present in the study area – despite its inability to resolve narrow band absorptions. We observe a tentative but consistent physical relationship between the gradation of substrate reflectance in the feature space and the gradation of sand versus clay content in the soil classification system.

## 1. Introduction

The availability of hyperspectral data for scientific purposes is rapidly increasing. The recent opening of the AVIRIS [1] and HICO [2] data archives offers scientists a wealth of high quality observations. Planned satellite missions such as HyspIRI [3], EnMAP [4], and HYPXIM [5] promise to offer even greater spatial and temporal coverage of narrowband imaging observations in the coming years.

These newly available and planned hyperspectral datasets complement the existing wealth of public multispectral satellite observations. The Landsat archive offers over 35 years of rigorously intercalibrated multispectral data [6]. The recent and planned launches of the Sentinel satellite constellation promise to add considerably to this collection [7], substantially decreasing the revisit time between subsequent multispectral observations [8].

The future availability of systematic global archives of both hyperspectral and short revisit time multispectral observations is expected to offer significant potential for studies that leverage both the spectral resolution of hyperspectral sensors and the temporal resolution of multispectral sensors. A growing number of data fusion techniques attempt to explicitly merge hyperspectral and multispectral data (e.g., [9,10]). More generally, hyperspectral imagery can be used to better understand fundamental properties of multispectral data. Regardless of the specific application, any combined use of hyperspectral and multispectral data will benefit from a better understanding of a fundamental question: How much of the spectral dimensionality of hyperspectral data is captured in multispectral data?

As first recognized by the pioneering work of Kauth and Thomas [11], multispectral observations generally form a three-dimensional pyramidal feature space. Subsequent global analyses of Landsat [12,13,14] confirm this finding on spatially extensive, spectrally diverse sets of data and show that these spaces generally contain >97% of their variance in the first three dimensions. Consistent global endmembers (EMs) can be identified from the apexes of the space representing soil and rock substrates, photosynthetic vegetation, and dark targets such as shadow and water. However, most analyses are regional and sample only a small fraction of the diversity of land covers present on the surface of the Earth. Another fundamental question is thus: Is the characteristic pyramidal structure of the multispectral feature space also present in the low order dimensions of the hyperspectral feature space at comparable spatial scales?

Finally, the majority of the EM variability observed in global multispectral analyses is in the substrate EM [11,14,15]. This is due to the wide range of soil and rock compositions that cover the land surface of the earth. It is likely that the global availability of hyperspectral imagery will add additional constraints to the properties of multispectral substrate EMs. A third fundamental question is thus: How much of the variability in Substrate EMs present in hyperspectral data is captured by multispectral sensors?

In this analysis, we use coincident AVIRIS and Landsat 8 acquisitions to address these three fundamental questions. We find the nearly identical partition of variance, low order feature space topology, and EM spectra for the hyperspectral and multispectral cases. The resulting feature spaces and EMs are also very similar to those found in previous global multispectral analyses, implying that the relatively simple pyramidal structure of the broadband feature space also represents the low order dimensions of the hyperspectral feature space. Finally, we find that the multispectral dataset well represents the basic structure of the plane of substrates found in the hyperspectral data, despite the fact that AVIRIS resolves a wide range of absorptions indistinguishable to Landsat OLI. Our results also suggest a novel, potentially useful method for mapping sand versus clay soil composition–from both hyperspectral and multispectral observations–on the basis of the location of soil EMs on the plane of substrates.

## 2. Background

We provide a background for each of the three central questions of the paper in order:

### 2.1. Measuring Spectral Variability

A reflectance spectrum is a characteristic of a material. Reflectance is the continuous, wavelength dependent function which describes the fraction of incident light which is reflected in a given direction. One profitable way of conceptualizing a Visible to Shortwave Infrared (VSWIR) reflectance spectrum is as a composite of two signals: the continuum and the absorptions [16]. Hyperspectral imagers generally oversample the reflectance spectrum, allowing for the analysis of both continuum and absorptions. Broadband instruments undersample the spectrum unevenly, often blurring together the continuum with the absorptions.

One way of quantifying the variability of reflectance (or radiance) vector sets is through their covariance matrix. For an image with *n* spectral bands, the spatial variance and covariance of each pair of bands forms an *n* square positive semi-definite matrix with *n* eigenvectors, representing uncorrelated modes of spectral variability, and *n* eigenvalues, representing the fraction of total variance described by each eigenvector and its corresponding spatial Principal Component, or PC.

Because of spatial and spectral redundancy in large numbers of reflectance measurements, the actual dimensionality of the spectral feature space may be less than the number of spectral dimensions provided by the sensor. The uncorrelated modes of spectral variability given by the eigenstructure of the covariance matrix generally contribute unequally to the total variance of the data. As a result, the most important modes (i.e., those contributing the most variance) can be used to represent the underlying structure of the spectral feature space. This provides an optimal representation of the diversity and interrelationships (e.g., mixing) of the most disparate spectral patterns in the data. In this context, spectral dimensionality refers to the distinction between the most informative dimensions, describing the physically meaningful structure of the spectral feature space, and the remaining dimensions that describe stochastic variance like sensor noise, atmospheric effects (or correction artifacts), and natural spectral variability with a less straightforward physical meaning than that represented by the lower dimensions.

This technique has been used to estimate the information content of hyperspectral [17,18,19,20] imagery, as well as to describe the information loss when reflectance spectra are undersampled by multispectral imagers [21,22]. Eigenvalues have also been used to directly quantify the dimensionality of multispectral data without reference to hyperspectral imagery [15]. Finally, the approach has also been used in the spatiotemporal analysis of image time series [23,24,25].

The key assumption behind the use of this approach to estimate data dimensionality is that variance directly corresponds to information. As noted by [18], this is a key limitation for its use with hyperspectral data because the location and depth of specific absorptions provide substantial information in a small amount of overall variance. Despite this limitation, we use this method in our analysis because it is a reliable, well-understood tool with both precedent and broad applicability. The purpose of this work is to present a comparative analysis of the characteristics of coincident hyperspectral and multispectral observations of a wide range of diverse land covers.

### 2.2. Spectral Variability and the Spatial Dimension

Image dimensionality is an attempt to measure the spectral diversity captured by a dataset. However, the spectral diversity of the Earth surface is spatially heterogeneous. The local variations that can exist in Earth surface reflectance, as well as the dimensionality of a large, diverse set of hyperspectral data, are well illustrated by the recent study of [19].

One might expect imagery with a higher spatial resolution to resolve more spectral diversity because of fewer mixed pixels. While this is generally true, the increase in spectral diversity (and dimensionality) with spatial resolution depends on the characteristic spatial scale of the reflectance of the landscape relative to the resolution of the sensor. This dependence of dimensionality on spatial resolution and SNR has long been recognized [22,26,27,28]. While it is not the focus of this study, it is noteworthy that the AVIRIS data we use have roughly half the Full Width at Half Maximum (and thus four times the spatial resolution) of the Landsat data.

All else equal, more spatially extensive image domains might also be expected to include a wider range of reflectance spectra and so have higher dimensionality—up to a point. As with spatial resolution, the dependence of spectral diversity on domain size depends on the characteristic spatial scale of the landscape reflectance. However, if the spectral diversity within an area approaches the global spectral diversity of the Earth, imaging a larger area should not add additional dimensionality.

The total area of land surface required to approach the level of global spectral diversity is clearly location dependent. However, the relationship between spatial resolution, extent of spatial domain, and the characteristic spatial scale of Earth’s spectral diversity is important because it determines which combination of extent and resolution may provide the most informative depiction of the spectral feature space as imaged by the sensor. This is important because the spectral feature space provides a physically-based depiction of the properties of a landscape that a given sensor is able to distinguish.

Part of the answer to this question lies in the overall global spectral diversity of the Earth. Previous work has attempted to address this. A global analysis of total Earth radiance as measured by the hyperspectral Scanning Imaging Absorption Spectrometer for Atmospheric Cartography (SCIAMACHY) was conducted by [29], finding 99.5% of variance explained in six dimensions. However, the spatial resolution of ~30 km attenuates much of the spectral variability of interest by spectral mixing. Due to the current absence of systematic global hyperspectral imagery of the land surface, no analog exists to these studies. The largest area study of Earth surface hyperspectral imagery to date was performed by [19]. This study used 15 m resolution AVIRIS imagery of a wide range of land cover types in California and estimated the overall spectral dimensionality at 50 dimensions. The broadband spectral dimensionality of a wide range of environments has been characterized for six-band multispectral Landsat imagery [13,14]. Because Landsat resolves only the spectral continuum, it can represent 99% of its spectral variability in only three dimensions. The structural similarities of the low order feature spaces in Boardman and Green’s 2000 analysis of 510 AVIRIS scenes [18] and Small’s 2004 analysis of 100,000,000 Landsat spectra [14] suggest that multispectral and hyperspectral feature spaces may share a common structure, despite the vastly greater information content of hyperspectral data. The analysis of 2.5 × 10^9^ AVIRIS spectra by Thompson et al. [19] suggests that the spectral diversity observable within California can represent all of the canonical land cover types included in the global MODIS land cover classification. In the present study, we use near coincident acquisitions of AVIRIS and Landsat 8 OLI to compare the spectral feature spaces and EMs in a pedologically diverse range of environments in the Great Central Valley of California.

### 2.3. Spectral Variability of the Plane of Substrates

Linear mixture models [30,31,32] are a common way to represent multispectral and hyperspectral data as linear combinations of spectral EMs. The three most common endmembers used for these models are soil and rock substrates, green vegetation, and dark or unilluminated materials (e.g., [33]). Non-photosynthetic vegetation is also commonly added as a fourth EM.

Of these EMs, the majority of the multispectral variability on a global scale is observed in the substrate [14,15]. This is due to the wide range of physical, chemical, biological, and textural properties present in soil, sediment, and rock. Reflectance spectroscopy of substrates is complex. Substrate reflectance spectra can vary in at least three ways: in continuum shape, in broad absorptions (e.g., Fe), and in narrow absorptions (e.g., some clays). Broadband imagery can only be expected to capture the coarsest of these features. For a comprehensive treatment of soil reflectance (a complex subset of the plane of substrates), see the seminal work of [34], more recent analyses by [35,36], and reviews by [37,38].

Early work on the hyperspectral dimensionality of the plane of substrates focused on full range Visible to SWIR (VSWIR) laboratory spectra [39] and Visible to NIR (VNIR) field spectra [40] of soils. Further laboratory VNIR characterization of a wide range of soils was performed by [41], although the dimensionality of the dataset was not discussed. The question of how the full diversity of hyperspectral VSWIR substrates (including soil, rock, and sediment) are represented in the spectral feature space, as well as how the signal is degraded when undersampled by a multispectral imager, remains open. The present study addresses this question by directly comparing substrate spectra from simultaneously acquired VSWIR hyperspectral and multispectral data, as well as by presenting a parallel analysis of 161 soil and rock laboratory spectra.

Portions of these three questions have been investigated previously. The questions of data dimensionality and generality of the feature space have been addressed independently for hyperspectral and multispectral datasets, but not simultaneously for both. Variability of the plane of substrates has been studied by the analysis of libraries of laboratory and field spectra. However, to our knowledge, none of these three questions have been addressed using simultaneous, independently imaged multispectral and hyperspectral observations over large areas of a spectrally diverse landscape.

## 3. Materials and Methods

**Figure 1:**
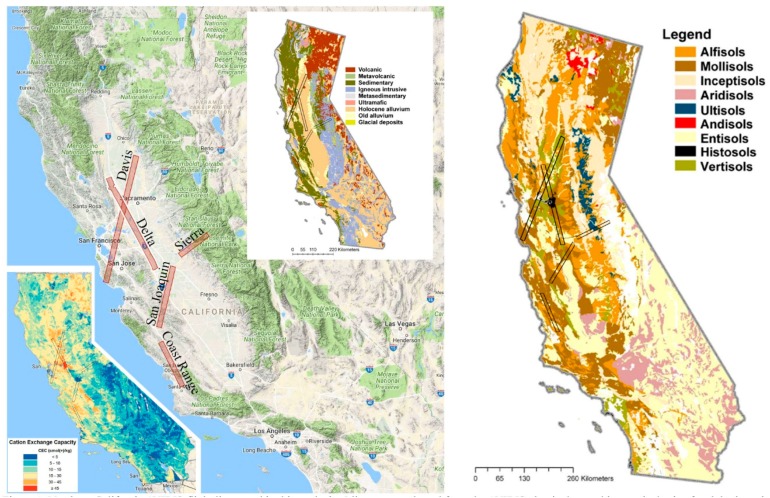
Northern California AVIRIS flight lines used in this analysis. Five lines were selected from the AVIRIS classic data archive on the basis of pedologic and agricultural diversity. Landsat 8 acquisitions were collected on the same day as each of the five flight lines. Simplified maps showing soil orders (right), as well as lithologic provenance (inset, upper right), and cation exchange capacity (inset, lower left) illustrates the complexity of soil properties in the study area. Six of the nine soil orders are represented in these flight lines. Soil maps from the UC Davis California Soil Resource Lab (https://casoilresource.lawr.ucdavis.edu/). Map data © 2017 Google INEGI.

Figure 1 shows the study area for this analysis in northern and central California, USA. This region was chosen because of the confluence of data availability from the recently opened AVIRIS hyperspectral image archive (https://aviris.jpl.nasa.gov/alt_locator/) and high-quality soil maps maintained by the UC Davis California Soil Resource Lab (https://casoilresource.lawr.ucdavis.edu/). The study area spans an unusually wide range of soil orders, textures, and chemical properties. A diversity of soil provenances is also present as a result of the geologic diversity of the region. Six of the nine soil orders in California are sampled in this study.

**Figure 2:**
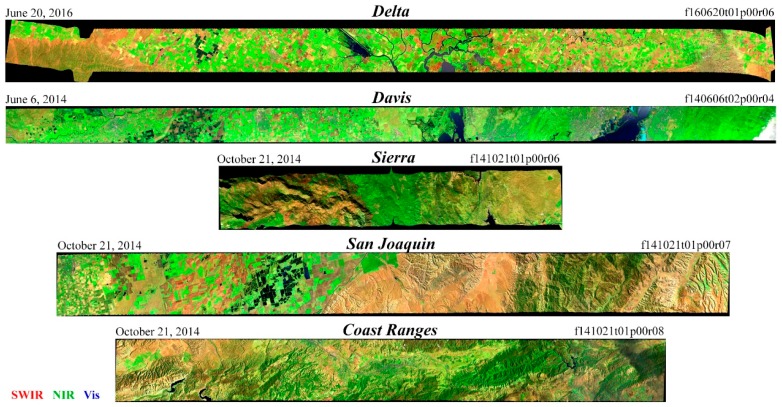
False color composite images of the five AVIRIS flight lines used in this study. A wide range of agriculture in the Great Central Valley of California is sampled in both summer and fall. Bare rock substrates are present in the Sierra and Coast Ranges. Non-photosynthetic vegetation (NPV) is present in both agricultural and natural land cover regimes. Cloud contamination near the ends of the Delta, Davis, and Coast Ranges lines was excluded from the remainder of the analysis. Wetlands are sampled in the Delta line and the San Joaquin line. Spectrally complex salt ponds are present in the South Bay portion of the Davis line. Sun glint is present in some water bodies.

Five AVIRIS flight lines were selected for use in this analysis on the basis of pedologic and spectral diversity (Figure 2). Critically, all five flight lines were acquired on the same day as coincident Landsat 8 acquisitions, allowing for a direct comparison of the effect of spectral resolution on dimensionality, as well as the opportunity for a direct comparison of AVIRIS and Landsat standard surface reflectance products. A diversity of agricultural and natural vegetation, settlements, bare soils, and water is present in the dataset. Some geological diversity is present in crystalline basement and sedimentary rock outcrops within the Sierra and Coast Range flight lines. Wetlands are present in the San Francisco Bay-Delta, as well as the San Joaquin River National Wildlife Refuge.

All AVIRIS images were downloaded as Level 2 Atmospherically Corrected Reflectance from the AVIRIS Data Portal at https://aviris.jpl.nasa.gov/alt_locator. Coincident Landsat 8 LaSRC modeled surface reflectance products were downloaded from the USGS at https://earthexplorer.usgs.gov.

**Figure 3:**
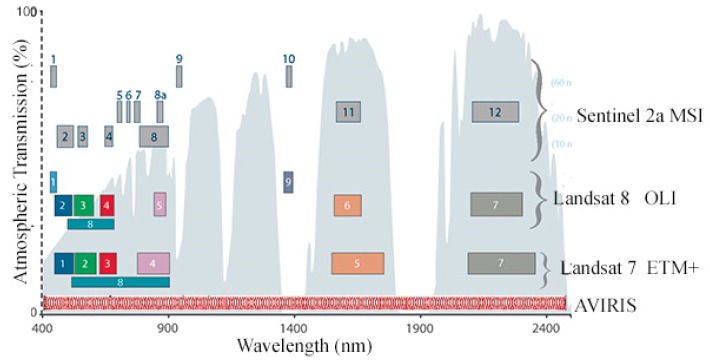
Spectral band comparison for Landsat 7/8, Sentinel 2, and AVIRIS. The AVIRIS hyperspectral sensor has 224 bands with 10 nm FWHM in the 365 to 2500 nm range. Spatial resolution of the AVIRIS IFOV depends on flight altitude. Modified from https://landsat.gsfc.nasa.gov.

Figure 3 shows a comparison of sensor resolutions for Landsat 8 OLI and AVIRIS, as well as the older Landsat 7 ETM+ and newer Sentinel 2 MSI sensors. The AVIRIS sensor images the Earth in 224 channels over the full VSWIR 365 to 2500 nm spectral range at a 10 nm spectral resolution. In comparison, multispectral sensors such as Landsat and Sentinel 2 collect a much smaller number of spectral bands with much wider bandpasses. As of the date of publication of [1], the signal-to-noise ratio (SNR) of AVIRIS was approximately 1000 for the VNIR, 700 for SWIR1, and 250 for SWIR2. In contrast, the Landsat 8 OLI SNR ranges between 201 and 367 for the bands used in this study [42]. AVIRIS oversamples most features in the reflectance spectrum and is therefore also able to resolve physical parameters about spectral slope and narrow band absorptions which are averaged together by OLI.

The five AVIRIS flight lines were subdivided into seven spatial subsets of 5000 × 700 pixels each. Coincident multispectral Landsat images were coregistered to match the hyperspectral subsets using nearest neighbor spectral resampling. All individual AVIRIS pixels were preserved without pixel averaging or interpolation. Because the spatial resolution of the AVIRIS data used in this study is somewhat higher (15 m to 17 m) than the 30 m resolution of Landsat 8, no direct pixel-to-pixel comparisons are attempted. Fortunately, the spatial scale of the land cover features of interest is generally coarse enough to allow a comparison of multipixel means of homogenous targets. The total areal coverage of the seven spatial subsets is approximately 9800 km^2^.

Bad and no data pixels present in either dataset were flagged and removed from the analysis. AVIRIS channels 108–133 (1363 to 1423 nm), 154–167 (1827 to 1927 nm), and 223–224 (2480 to 2500 nm) were excluded from subsequent analysis because of a large number of pixels with nonphysical reflectance values (i.e., high amplitude positive and negative spikes). Landsat 8 OLI coastal aerosol and cirrus bands (1 and 9) were excluded from this analysis because land cover is the focus of this study. The Landsat 8 panchromatic band was not used.

Image statistics were then computed for the remaining 181 channels of the AVIRIS image and PC rotations of all the AVIRIS spectra were computed based on both the image covariance and correlation matrices. Eigenvalues were normalized by their sum to compute the fraction of variance present in each dimension. The same procedure was repeated for the six band Landsat 8 pixels. A Minimum Noise Fraction (MNF) transform [43] was also used for comparison, but yielded similar EMs to the PC-derived feature space.

For comparison with pure substrate EMs, 161 laboratory spectra of rocks and soils from the Johns Hopkins University (JHU) spectral library were used for a PC analysis, as described above. For more details on the JHU spectral library, see: https://speclib.jpl.nasa.gov/documents/jhu_desc.

## 4. Results

**Figure 4:**
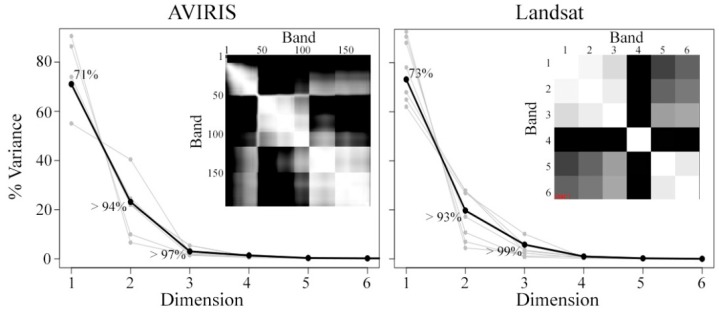
Partition of variance for AVIRIS (**left**) and Landsat (**right**). Result are shown for each 5000 × 700 pixel spatial subset (gray), as well as for the combined dataset (black). While the dimensionality of each spatial subset is variable, the combined dataset shows the first three dimensions clearly separated from the continuum that follows. Cumulative variance is labeled for the first three dimensions, showing that 97% or more of the total variance in contained in the first three dimensions of each dataset. Correlation matrices (inset) clearly show three or four distinct spectral regimes which capture the majority of the variance in the dataset.

Figure 4 shows the low order partition of variance for each of the 5000 × 700 pixel AVIRIS subsets (light gray), as well as the full 5000 × 4900 pixel image composite (black). While some variability is observed in the partition of variance for individual spatial subsets of both the AVIRIS and Landsat datasets, the first three or four dimensions clearly contain nearly all of the variance in all cases. Cumulative variance for the first three dimensions of the AVIRIS and Landsat composite images is 97% and 99%, respectively. As expected, inset correlation matrices illustrate three or four relatively distinct spectral regimes (Visible, NIR, SWIR1, and SWIR2). A more complex correlation structure is evident within the AVIRIS NIR spectral regime, illustrating the added value of the hyperspectral sensor.

**Figure 5:**
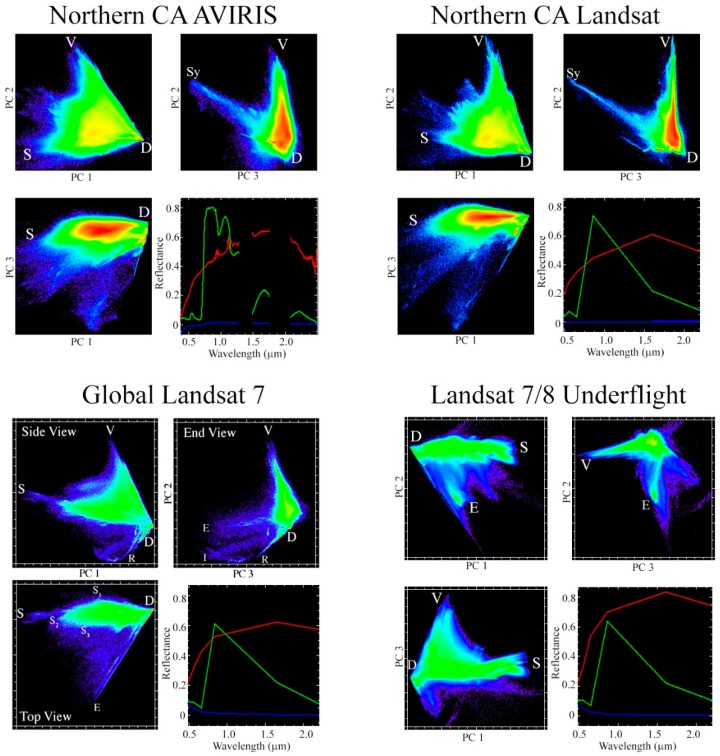
Low dimensional feature space comparison. Northern CA AVIRIS and Landsat used in this study both show similar low-order feature space topology to the much wider range of land covers sampled in the global Landsat 7 analysis of Small & Milesi (2013), as well as the Landsat 7/8 underflight comparison analysis of Sousa & Small (2017). Substrate (S), Vegetation (V), and Dark (D) EMs are also broadly consistent across all four studies, in spite of the substantial disparities in both spectral resolution and spatial extent. Differences in topology are predominantly due to variations in sampling of relatively rare land covers such as evaporites (E) and synthetic materials (Sy). Substrate EMs show the greatest variability, as expected given the diversity in the shape of the reflectance continuum of rock and soil.

Figure 5 shows projections of the first three dimensions of the spectral feature spaces for the AVIRIS and Landsat datasets of this study (top) in comparison to the much more extensive spatial sampling of previous studies by [12,13,14]. The first three dimensions of the AVIRIS and Landsat feature spaces show striking similarity to each other, despite the roughly 30× greater spectral sampling of the AVIRIS. Both covariance- and correlation-based transforms were performed. Because the resulting feature spaces were nearly identical, only the feature space from the covariance-based transform is shown here.

In addition, both of the feature spaces of this study are remarkably similar to the global Landsat feature spaces. This observation is especially noteworthy given the differences between the studies in data preprocessing. Substrate, Vegetation, and Dark EM spectra show comparable spectral shapes despite the relatively minimal sampling of crystalline basement or large scale sedimentary deposits.

The most prominent differences between the feature spaces of this study and previous studies involve the sampling of evaporites and synthetic materials. This study does not sample any large deposits of evaporitic minerals such as Halite or Gypsum. These minerals are spectrally distinct from other soil and rock substrates and they plot separately from the main point cloud that represents the vast majority of land surface reflectance spectra. This study does, however, contain a relatively large fractional area of settlements with synthetic roofs and tarped fields. These relatively exotic spectra do not have appreciable global abundance and so are not present in the previous studies focusing on terrestrial targets.

Multispectral green vegetation and dark EMs are generalizable globally with spectral shapes consistent with physical and biological properties. However, as has been noted in numerous previous studies (e.g., [16,17,18]), rock, sediment, and soil substrates demonstrate a diversity of spectral shapes. Local substrate EMs are generally used for more accurate spectral unmixing results because they can take into account this regional pedologic and geologic diversity. These EMs can be selected directly from the spectral feature space.

For comparison with pure substrate EMs spanning a wider range of rocks and soils than that found in the study area, we rendered a spectral feature space from laboratory spectra of rock and soil samples. Using the Johns Hopkins University (JHU) spectral library, we compiled 161 laboratory rock and soil reflectance spectra. The spectra span a wide range of soil and rock types, including 67 igneous rocks, 55 metamorphic rocks, 14 sedimentary rocks, and 25 soils.

**Figure 6:**
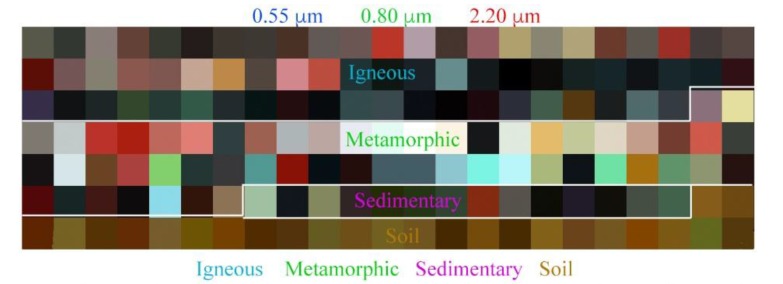

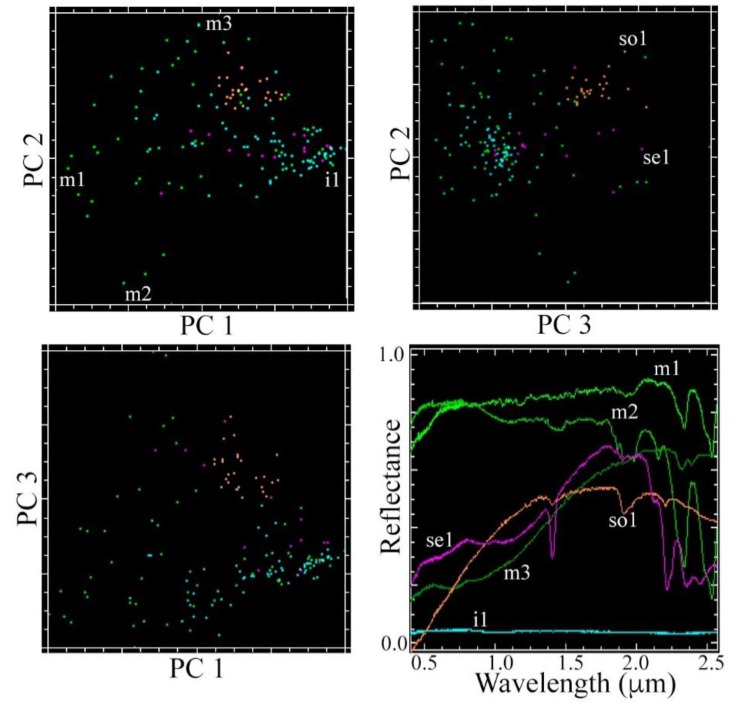
Spectral feature space and endmembers for the rock and soil substrate spectra from the JHU spectral library. Visible/NIR/SWIR false color composite (**top**) gives some indication of the spectral diversity in three principal wavebands. Three orthogonal projections of the spectral feature space (**bottom**), along with example endmember spectra from the periphery of the space, illustrate the broad diversity of metamorphic spectra compared to the more continuous soil spectra.

PCA of the JHU substrate spectra reveals the first three dimensions account for 85.8%, 10.5%, and 2.2%, respectively. The first three dimensions thus account for over 96% of the overall variance of this diverse library of substrate spectra. The spectral feature space in Figure 6 shows that the 25 soils cluster in a relatively confined subset of this substrate space. The soil spectra are observed to deviate from each other in much less pronounced ways than rocks vary both within and between the igneous, metamorphic, and sedimentary classes. Given the dominance of agricultural land cover in our study area, it is this relatively narrow range of soil spectra which we would expect to dominate as the substrate EM in our Landsat-AVIRIS analysis.

**Figure 7:**
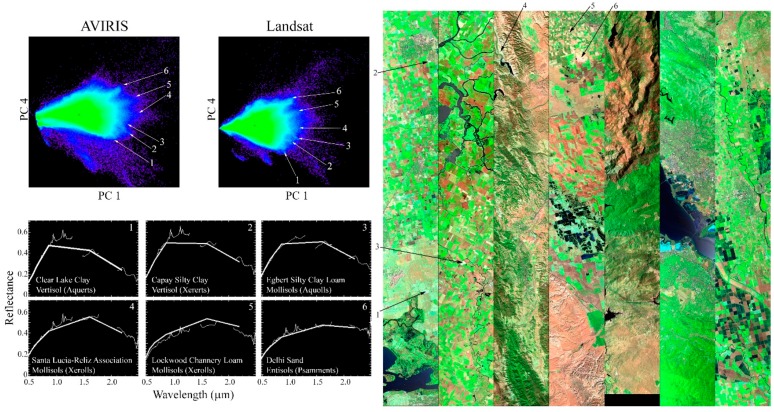
AVIRIS and Landsat substrate EM variability. The PC 1 vs. 4 projection is useful in both transforms as a means of discriminating between the spectral shape of substrate EMs. The remarkable topological similarity between the AVIRIS and Landsat feature spaces (**upper left**) extends into the fourth dimension. The bright (high PC1) edge of the point cloud splays out in both AVIRIS and Landsat to reveal a continuum of rock and soil substrate EM spectra. Multipixel mean spectra for each of these EMs (**lower left**) are displayed as observed by both Landsat and AVIRIS. The locations of the spectra are indicated on the AVIRIS flight lines (**right**). USDA-NCSS soil survey data were used to find the soil type at the location of each EM. The order and suborder of each soil are shown on the EM spectra plots. The spectral variability captured by the EM continuum corresponds to consistent soil property variations. From EM1 to EM6, the soil types are characterized by a tradeoff between decreasing clay content and increasing sand content.

The range of substrate EMs actually present in this analysis is shown in Figure 7. In order to clearly identify these substrate EMs, the fourth dimension of the spectral feature space is used. While accounting for less than 2% of the total variance in either dataset, PC 4 proves to be useful in this case for distinguishing between the shapes of soil and rock spectra.

Six substrate EMs are identified from the PC 1 vs. 4 projection of the feature space. The clear visual similarity in feature space topologies and EM spectra between the hyperspectral and multispectral datasets continues through the fourth dimension. This similarity is unsurprising given the dominance of the spectral continuum (rather than narrowband or broadband absorption features) in the reflectance spectra of these soil EMs.

Each EM corresponds to a spatial cluster of points in a bare (or senescent) agricultural field or a spatially extensive sedimentary deposit. The locations of the EMs are indicated on both the feature space and the false color composite of the flight line subsets. USDA-NCSS soil survey data were acquired for the location of each spectral EM using the California Soil Resource Lab SoilWeb browser (https://casoilresource.lawr.ucdavis.edu/gmap/). The map unit name is given for each EM (e.g., Clear Lake Clay), as well as its order (e.g., Vertisol) and suborder (e.g., Aquerts).

Notably, the spectral properties of the continuum of substrate EMs appear to correspond to a continuum of soil grain sizes and textures. The soil at EM 1 is classified as a Clay, with surface composition of the dominant series (Clear Lake) of roughly 60% clay and 15% sand. In contrast, the soil at EM 6 is classified as a Sand with typical surface composition of its dominant soil series (Delhi) of roughly 4% clay and 95% sand. EM 3 is classified as a Silty Clay Loam, and the surface composition of its dominant series (Egbert) lies in between, with 38% clay and 18% sand fractions. While preliminary, these results suggest that some soil compositional properties may be sufficiently spectrally distinct to be mapped using their position in the feature space–at least in this study area.

**Figure 8:**
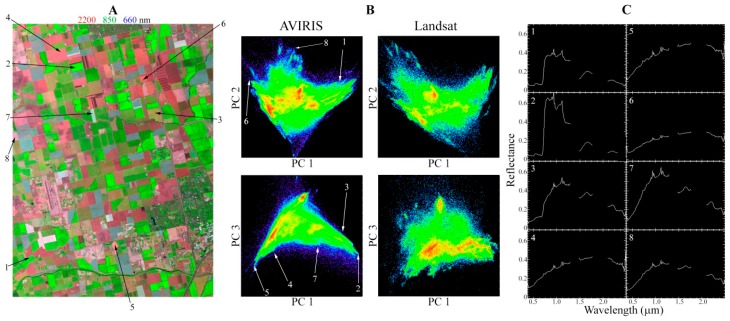
Spectral diversity in a 9 × 15 km region of intensive agriculture in the Great Central Valley of California. Considerably more spectral diversity is present in this hyperspectral dataset than can be adequately represented by any false color composite image Locations of spectra a-h are shown on the AVIRIS feature space and on the image. **(A)**. The AVIRIS feature space for this spatial subset (**B**, **left column**) clearly shows more prominent clustering than the Landsat feature space (**B**, **right column**). Green fields are characterized by a wide range of red edge and absorption properties (**C1**,**C2**). Bare or senescent fields are characterized by an even greater range of spectral shapes (**C3**–**C8**). This high concentration of spectral diversity is consistent with the similarity in dimensionality and feature space topology between the limited area of this study and the much more extensive global studies of Small & Milesi (2013) and Sousa & Small (2017).

Figure 8 illustrates the complexity that can be measured by hyperspectral imagery on even a relatively small spatial scale. The spatial subset shown in this figure is only 9 × 15 km. Even this small spatial area exhibits considerable diversity in vegetation and substrate spectra. Substantially more prominent clustering is notable within the AVIRIS feature space than within the Landsat feature space, as the subtle spectral differences between fields are better resolved by the hyperspectral sensor than the multispectral sensor. The full complexity of these spectra could not be captured in any three band false color composite image, or resolved with the six-band Landsat or even 11-band Sentinel-2 sensors. Individual pigment and mineral absorptions are distinguishable that would be lost completely in multispectral data. Notably, despite the severely limited spatial domain of this subset, spectra are identifiable (e.g., 2 and 5) that are again remarkably similar to global EMs.

## 5. Discussion

This work addresses three questions:

(1) How much of the dimensionality of hyperspectral data is captured in multispectral data?
(2) Is the characteristic pyramidal structure of the multispectral feature space also present in the low order dimensions of the hyperspectral feature space at comparable spatial scales?
(3) How much variability in Substrate EMs present hyperspectral data is captured by multispectral sensors?

The first question of this study is addressed quantitatively by the hyperspectral and multispectral partitions of variance given in Figure 4. It is further addressed qualitatively through the topologies of the low-order feature spaces shown in Figure 5 and Figure 6. The spectral feature spaces show a striking similarity in the dimensionality, topology, and EM spectra of the coincident hyperspectral and multispectral datasets used in this study. This is observed despite a factor of 4 difference in the sensor spatial resolution, a factor of 30 difference in the spectral resolution, and further differences in atmospheric correction procedures between the products. At least 97% of the variance in each dataset is present in the first three dimensions and >99% is present in the first four dimensions for both sensors.

This result is in accord with the multispectral findings of [14]. It suggests a substantially lower dimensionality than the previous hyperspectral studies of [19,27,28], but this is because our metrics for dimensionality differ. A primary purpose of dimensionality estimation by these previous AVIRIS studies was to quantify the number of unique materials that the hyperspectral sensor can image above noise level. This is not our purpose. Rather, we seek to describe the number of independent dimensions in which the majority of the spectra reside when decomposed by variance, the number of EMs which bound the space, and the way in which these EMs trade off. This is a fundamentally different question and is why we examine the topology of the point cloud so closely in addition to the partition of variance.

The information content of a dataset is not a trivial quantity to estimate. We are in full accord with [18] in the opinion that “measuring dimensionality in multivariate data sets is a slippery slope and an approximation at best”. We also agree with the findings of [19], that the overdetermined nature of hyperspectral imaging “is highly desirable since it offers numerical leverage while also providing the capability to measure unexpected phenomena and falsify modeling assumptions”. It is additionally clear that much of the value of hyperspectral data resides in the precise measurement in the location, depth, and breadth of absorption features. This analysis shows that these features are not well-represented in dimensionality estimates given by the PC transform, nor by deviations in the topology of the point cloud, at least in the first four dimensions. Variance is an imperfect metric for information content, especially in hyperspectral imagery.

While difficult to estimate, the question of dimensionality is important because it places in context the aggregate landscape-scale measurements of a sensor. The observation of the similarity of the multispectral and hyperspectral feature spaces suggests that, even with hyperspectral observations, the shape and amplitude of the spectral continuum dominates the variance structure of the data, but does not fully determine its information content. It is upon the structure of this low-order spectral feature space that the narrowband absorptions and other fine spectral features are superposed. Our results suggest that these information-rich fine spectral features do not appreciably change the fundamental low-order structure of the feature space. This finding has potential implications for the future synergistic use of multispectral and hyperspectral data.

**Figure 9:**
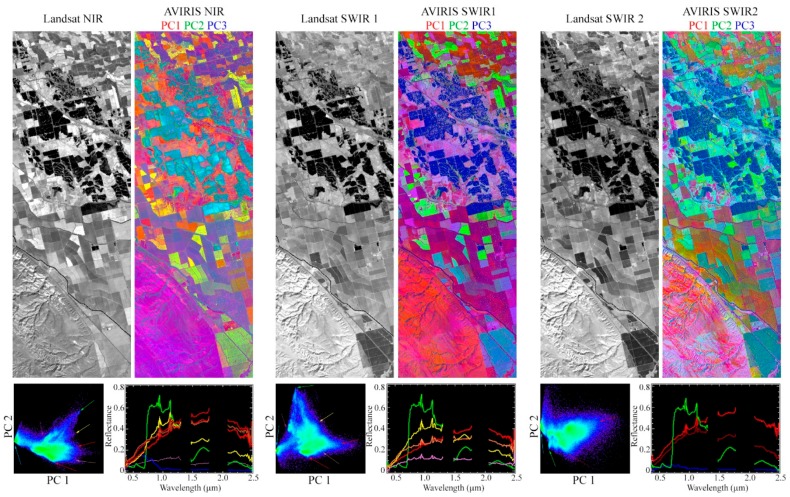
Intraband and interband spectral dimensionality of AVIRIS in comparison to individual infrared bands of Landsat OLI. OLI is one dimensional in each of the NIR, SWIRl, and SWIR2 spectral regimes because the sensor only collects in one band for each. In contrast, AVIRIS collects in over 40 channels for each of the three spectral regimes. The spectral dimensionality of the hyperspectral imagery is apparent in the color images of the three lowest-order Principal Components for the channels within each regime, as well as in the structure for the corresponding spectral feature spaces. Dark and vegetation EMs are similar for each feature space but substrate EMs differ because of distinct absorptions. EM locations are indicated on each feature space by colored arrows.

Figure 9 shows an additional way of visualizing information content present in these fine spectral features. In this figure, each of the NIR, SWIR 1, and SWIR 2 Landsat bands are shown individually for a single spatial domain. Because there is only one band for each of these spectral regimes, the Landsat dimensionality for each spectral subset is 1 and must be shown as a grayscale image. However, over 40 AVIRIS channels are present for each of these spectral regimes. Adjacent to each Landsat band is a tricolor composite showing the three low order PC images for the AVIRIS spectral subset corresponding to each spectral regime. Substantially greater information is obviously present in each spectral regime for the hyperspectral cube than the multispectral dataset. The dimensionality of AVIRIS is clearly at least two in each of these spectral regimes, yielding intraband feature spaces with a considerable structure and physically meaningful EMs.

The results of this study do not imply that the AVIRIS data cube only images three or four spectrally distinct quantities, nor that the information contained in the hyperspectral cube can be captured by a multispectral instrument. Rather, they demonstrate that, when decomposed linearly using the PC transform, the variance of both datasets is partitioned into a nearly identical number of fundamental dimensions and the relationship between the spectra follows a similar geometric relationship. It might have been expected that substantially different EMs would arise from the differences in spatial and spectral resolutions of the sensors. This was not the case. Furthermore, it might have been expected that a low-order dimension would emerge in the hyperspectral dataset which differed substantially from the multispectral dataset. This was not the case either. Rather, despite the significant variations in absorption features–most notably in the plane of substrates–the EMs were arranged in a nearly identical configuration. The implications of this result for multitemporal analyses are substantial, as they suggest that pixel trajectories in multispectral feature space correspond to nearly identical trajectories in low-order hyperspectral feature space.

The second question of this study is addressed quantitatively through the partitions of variance in Figure 4 and qualitatively by the feature spaces in Figure 5 and Figure 6. Both the hyperspectral and multispectral partitions of variance, feature spaces, and EMs found in this study are remarkably similar to those found in previous analyses of Landsat data sampling much more spatially extensive regions. These results suggest that a substantial fraction of global multispectral diversity can be sampled in a local spatial extent. Notably, the entire area used in this analysis, 9800 km^2^, is roughly 1/3 of the 34,000 km^2^ area covered by a single Landsat scene and less than 0.01% of the ice-free global land area. Similar EM spectra were even present within the very small 180 km^2^ region of Figure 7.

Finally, the third question of this study is directly addressed by Figure 6. Not only are the relative positions of the substrate EMs nearly identical, a consistent physical relationship is also observed between their position in the PC 1 vs. 4 projection of the feature space and the soil properties of the locations from which they are derived. This was observed in both the hyperspectral and multispectral datasets using independent rotations. While there is clearly more information to be gained from hyperspectral imagery than the topology of the point cloud, it is important that the multispectral and hyperspectral sensors distinguish between the same broad soil features in the same way. The similarity of the spectral feature spaces suggests significant potential for inferring properties of full soil reflectance spectra from multispectral observations. As indicated by Figure 6, this is not true for rock spectra in general. While soil spectra have generally similar shapes, rock spectra often have very distinct narrowband absorptions related to the crystal structure of specific minerals not generally preserved in soils. This consistency in the structure of the plane of substrates has the potential to substantially inform analyses in studies where a temporally or spatially sparse set of hyperspectral observations complement a wealth of multispectral observations.

Variability in grain size and textural properties of the underlying soils is a physically plausible explanation for the spectral variability observed in the feature space. The potential for soil-specific information in PC 4 has been previously documented by [15], but was only briefly mentioned and was not tied directly to specific compositional properties. To our knowledge, the question has not yet been further elucidated. Although our results for this subject are preliminary, as this was not the primary focus of the study, they are encouraging. One critical complicating factor moving forward will be controlling for the presence of non-photosynthetic vegetation.

While beyond the scope of this study, further investigation of the relationship between soil type and reflectance presents an attractive avenue for future work. In a pedologically diverse region such as California with quality soil maps and abundant hyperspectral observations, the potential exists to use hyperspectral feature spaces to determine which soil properties can and cannot be reliably determined from hyperspectral and multispectral imagery. The range of reflectance spectra corresponding to each soil class could be documented and a repeatable, systematic classification system (expanding upon the Munsell color chart) could potentially be developed on the basis of reflectance spectroscopy. The tools of the visible could be extended into the infrared.

## 6. Conclusions

We analyze the spectral dimensionality of five hyperspectral flight lines and coincident multispectral satellite images over a region of considerable pedologic and agricultural diversity. The partition of variance, spectral feature spaces, and EM spectra for each dataset bear remarkable resemblance to each other. Comparable similarity with earlier global multispectral analyses is also observed. These results demonstrate (1) that the multispectral and hyperspectral feature spaces share a fundamental low order structure, and (2) that the global multispectral feature space can be reasonably represented in a relatively small spatial domain.

In addition, a nearly identical continuum of substrate EMs is observed in both the multispectral and hyperspectral datasets. Comparison with a soil map shows that variability in soil composition strongly covaries with the position of EMs in the feature space. Our (local) success in discriminating between soil classes with variable sand vs. clay fractional compositions suggests considerable potential for a novel method for improving the mapping of soils with optical remote sensing.
